# Supplementary material for: Mechanomimetic 3D Scaffolds as a Humanized In Vitro Model for Ovarian Cancer
Source: Cells. 2022 Feb 26;11(5):824. doi: 10.3390/cells11050824 (PMC8909508; doi:10.3390/cells11050824)
Supplement: Supplementary file 1 [file cells-11-00824-s001.zip › cells-1604735-supplementary.pdf]

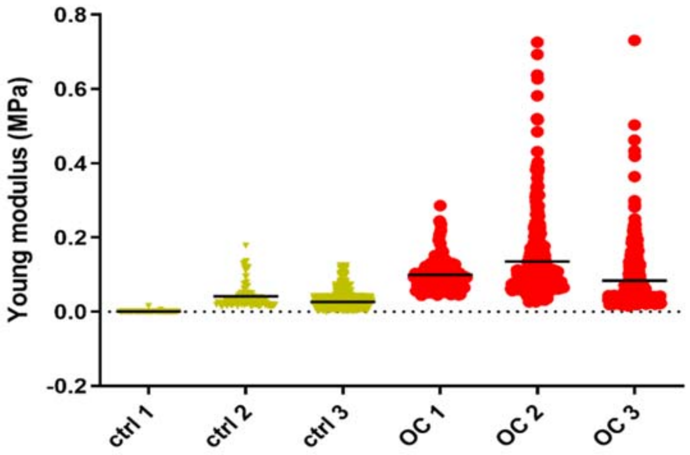

Figure S1. AFM analysis of patients' derived biopsies. (A) Young's modulus (MPa) analysis of 3 HGCS III (OC1-2-3) and 3 normal patient-derived biopsies (ctrl 1-2-3) by AFM.

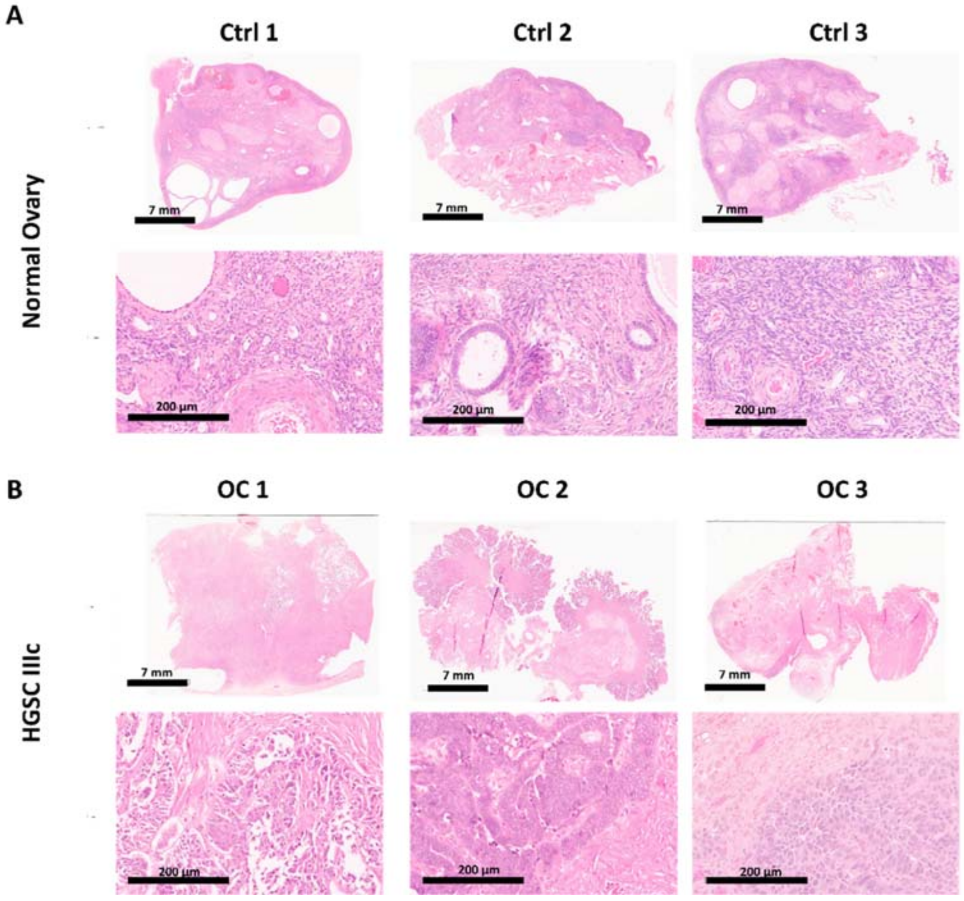

Figure S2. H&E characterization of patients' samples. (A) H&E staining of 3 normal ovary biopsies samples. (B) H&E staining of 3 HGSC IIIc biopsies samples. Images were acquired with 4x and 20x objective.

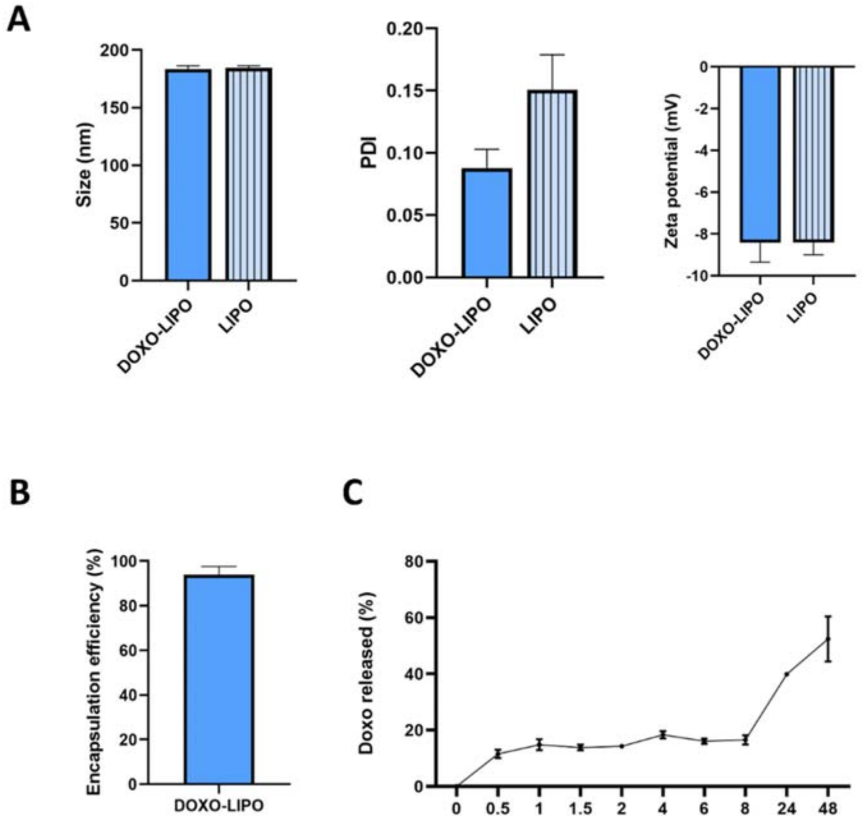

Figure S3. Physical and pharmaceutical characterization of empty (LIPO) and doxorubicin-loaded liposomes (DOXO-LIPO). (A) Dynamic light scattering and zeta potential measurements comparing empty LIPO and DOXO-LIPO. (B) Encapsulation efficiency and (C) release profiles of DOXO-LIPO in PBS +10% FBS (50:50) at 37 °C. Data are mean + standard deviation (n=3).

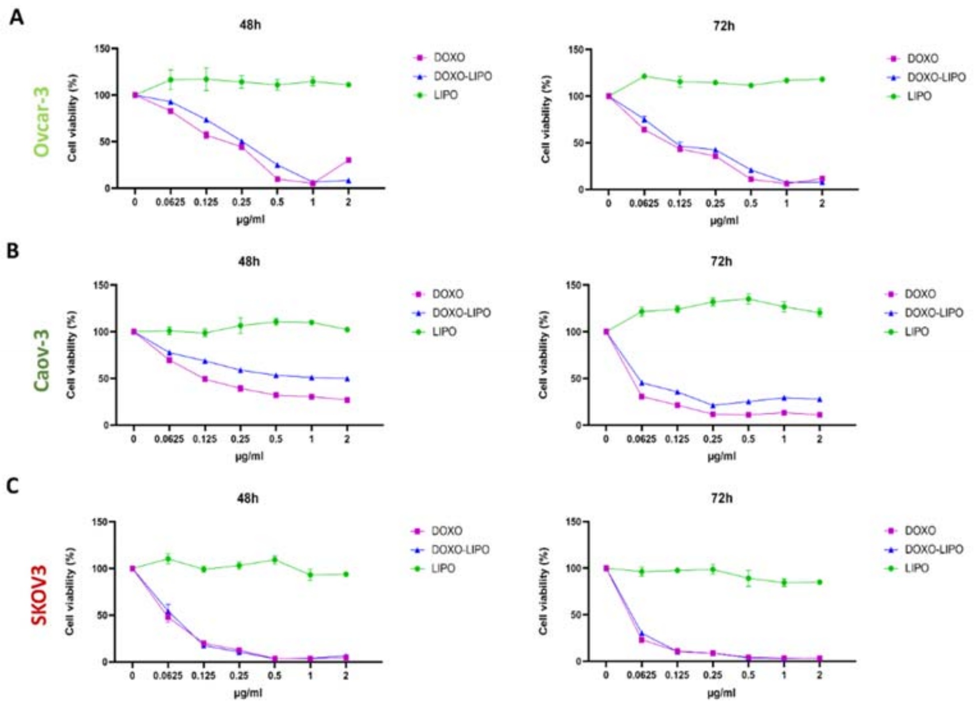

Figure S4. DOXO free and DOXO LIPO cytotoxic effect on ovarian cancer cell lines in 2D after 48h and 72h of treatment. MTT analysis after 48h and 72h of DOXO free and DOXO-LIPO treatment for OVCAR-3 (A), Caov-3 (B), SKOV3 (C) ovarian cancer cell lines. Data are mean + standard deviation (n=3).

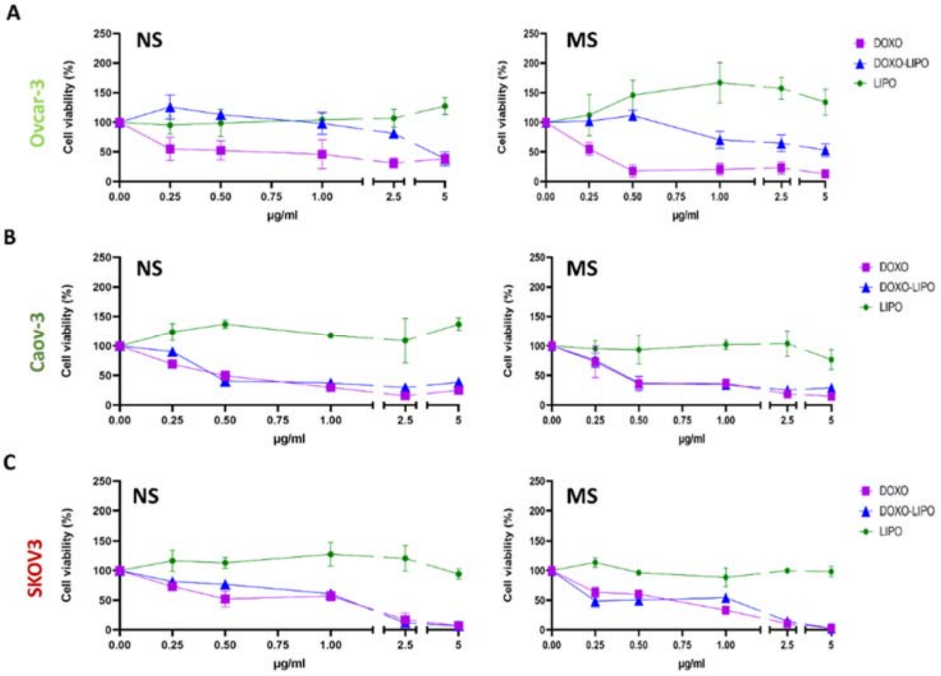

Figure S5. DOXO free and DOXO LIPO cytotoxic effect on ovarian cancer cell lines in 3D MS and NS after 72h of treatment. Cell TiterGlo analysis on cell viability under DOXO free, DOXO-LIPO and LIPO treatment for OVCAR-3 (A), Caov-3 (B), SKOV3 (C) ovarian cancer cell lines. Data are mean + standard deviation (n=3/5).
